# Identification of Spectral Modifications Occurring during Reprogramming of Somatic Cells

**DOI:** 10.1371/journal.pone.0030743

**Published:** 2012-04-13

**Authors:** Christophe Sandt, Olivier Féraud, Noufissa Oudrhiri, Marie Laure Bonnet, Marie Claude Meunier, Yannick Valogne, Angelina Bertrand, Martine Raphaël, Frank Griscelli, Ali G. Turhan, Paul Dumas, Annelise Bennaceur-Griscelli

**Affiliations:** 1 SOLEIL Synchrotron, Saint Aubin, Gif sur Yvette, France; 2 Inserm UMR 935, “ESTeam Paris Sud,” Stem Cell Core Facility Institut André Lwoff, University Paris Sud 11 Paul Brousse, Villejuif, France; 3 AP-HP Laboratory of Hematology, CHU, Bicêtre, France; 4 Inserm UMR 935, University of Poitiers, Division of Laboratory Hematology and Oncology, CHU Poitiers, Poitiers, France; 5 University Paris Descartes, Paris, France; Instituto Butantan, Brazil

## Abstract

Recent technological advances in cell reprogramming by generation of induced pluripotent stem cells (iPSC) offer major perspectives in disease modelling and future hopes for providing novel stem cells sources in regenerative medicine. However, research on iPSC still requires refining the criteria of the pluripotency stage of these cells and exploration of their equivalent functionality to human embryonic stem cells (ESC). We report here on the use of infrared microspectroscopy to follow the spectral modification of somatic cells during the reprogramming process. We show that induced pluripotent stem cells (iPSC) adopt a chemical composition leading to a spectral signature indistinguishable from that of embryonic stem cells (ESC) and entirely different from that of the original somatic cells. Similarly, this technique allows a distinction to be made between partially and fully reprogrammed cells. We conclude that infrared microspectroscopy signature is a novel methodology to evaluate induced pluripotency and can be added to the tests currently used for this purpose.

## Introduction

The last decade of stem cell research has witnessed paramount milestones from the first description of human ESC (hESC) [1] to the recent reprogramming techniques of adult stem cells [2]. hESC exhibit similarities with iPSC in terms of pluripotency and differentiation potential [2],[3]. However, despite their pluripotency, iPSC might not be entirely similar to hESC, especially with regard to their functional status. Indeed, a recent study has shown a gene signature and micro-RNA signature of iPSC distinct from that of hESC [4], [5]. Recent data reveal also an epigenetic memory preserved in iPSC according to the somatic cell of origin which will hinder or facilitate their differentiation as a function of this memory [6], [7]. Pluripotency is assessed directly by phenotypic criteria or indirectly by functional assays such as teratoma induction, or by global genomic approaches. However, methodologies allowing their identification using biophysical characteristics of the cells are lacking.

**Figure 1:**
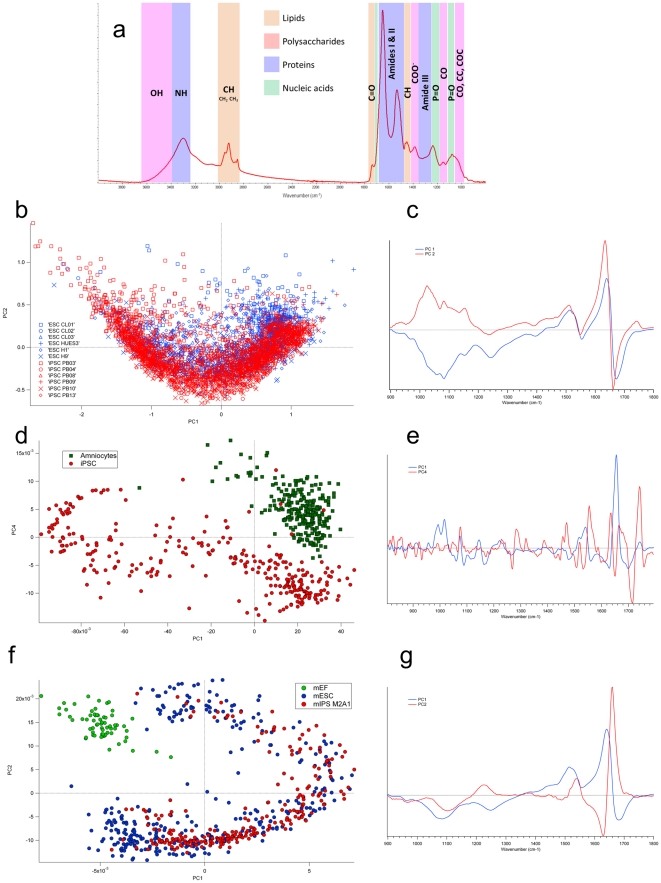
Analysis of infrared spectra obtained from human and murine ESC as compared to human and murine iPSC and the corresponding somatic cells. (A) Representative infrared spectrum obtained by FTIR microscopy. The infrared spectrum of a cell represents an integrated signature of the cell defined by chemical composition and metabolism that gives rise to a biochemical fingerprint of the cell identity. (B) Comparison of iPSC and non-isogenic hESC lines by Synchrotron FTIR microscopy. Six ESC (H1, H9, HUES3, CL01, CL03, CL04) (blue) and six unrelated iPSC cell lines (PB03, PB04, PB08, PB09, PB10, PB13) (red) were compared by MVA. The scoreplot based on PC1 and PC2 of Principal Component Analysis (PCA) shows that iPSC and ESC spectral signatures are very similar. (C) Loading plot corresponding to the comparison of iPSC and hESC. PC1 and PC2 show that the dispersion of iPSC and ESC spectra is related to changes in protein∶nucleic acid, protein∶glycogen contents, and in the proteome. (D) Comparison of spectral signatures of human somatic amniotic fluid cells (AFC) and their iPSC derivatives. Two independent AFC populations (blue) and their derived iPSC (PB03, PB04) (red) were compared. As can be seen in Scoreplot of the Principal Component Analysis AFC and iPSC derived from them could be differentiated by their spectral signatures. (E) Loading plot corresponding to the comparison of AFC and their iPSC derivatives. PC1 and PC4 allow separating AFC and iPSC spectra from changes in the lipid signal (1740, 1710, 1465, 1455, 1170 cm^−1^), in the proteome signal (1650, 1635, 1550 cm^−1^), and in the cellular phosphorylation (1270 cm^−1^, 1074 cm^−1^). (F) Comparison of spectral signature of MEF with the M2A1 iPSC and murine ESC. Scoreplot of the PLS-DA show a distinct and separated spectra of MEF (green), M2A1 (red) and 4 murine ESC (CJ7, R2, D3 and GS2) (blue). M2A1 clustered with the murine ESC. (G) Loading plot corresponding to the comparison of murine iPSC and mESC. PC1 and PC2 explaining the spectral differences between murine stem cells and MEF in the proteome and nucleic acid ranges.

In this work, we explored the chemical and metabolic components of iPSC and ESC by using the Fourier Transform Infrared (FTIR) spectroscopy, combined with a high brilliance synchrotron radiation source [8]. Synchrotron infrared microspectroscopy is a promising technique for biomedical and biological studies at subcellular resolution allowing the analysis of several cell components such as polysaccharides, nucleic acids, protein and lipid contents in terms of the spectra they generate (Fig. 1A and Table S1) while providing an excellent spectral quality, an important prerequisite for post-statistical analysis [8]. Importantly, this description can be obtained at single cell level and thus offers a powerful technique to explore the cellular diversity within a cell population.

## Results

We first explored a series of human iPSC that were generated from somatic amniotic fluid cells (AFC). All of these iPSC (PB03 to PB13) were validated for their pluripotency and have been registered on the European registry (www.hesreg.eu). Using FTIR microspectroscopy, we could not identify significant differences between 6 of these iPSC and 6 human ESC lines (Fig. 1B).

We noticed that the iPSC cloud is slightly shifted along the PC2 axis indicating that iPSC accumulate more glycogen, and have a slightly more active metabolism than the hESC lines. We performed a multivariate prediction approach in order to confirm that ESC and IPS cells are spectrally extremely similar. For such a purpose, we used a PLS-DA model with a calibration set containing more than 4000 spectra of ESC. A validation set of 505 spectra not included in the calibration set, was used for prediction, using 6 factors explaining 98% of the spectral variance. The PLS-DA prediction was evaluated through the computed sensitivity, specificity and Matthews correlation coefficient (MCC). The results, reported on Table S2 indicate that the prediction model was only slightly better than the performance of a random classifier (MCC 0.48) and correctly identified IPSC (sensitivity 83%) but failed to identify correctly ESC (specificity 63%). The prediction accuracy relied only on the 5^th^ and 6^th^ factors each accounting for less than 1% of the spectral variance but carrying 40% of the class difference. This confirmed that ESC and IPS cells are spectrally extremely similar. We then wondered whether we could identify any differences in terms of infrared spectra between the parental somatic AFC and their iPSC derivatives. As can be seen in Fig. 1D, AFC were found to exhibit a significant and distinct spectral signature from that of iPSC, suggesting that the reprogramming process modified significantly the metabolic composition of these differentiated cells. A PLS-DA model separated the AFC and IPS spectra from a validation set with a MCC of 1, a perfect prediction (Table S2). The differences were best seen using the loadings of the Principal Component Analysis (PCA). The main differences arise from the lipid composition and cellular concentrations (1740, 1710, 1465, 1455, 1170 cm^−1^), from changes in the proteome (1650, 1635, 1550 cm^−1^), and from changes in the cellular phosphorylation (1270 cm^−1^, 1074 cm^−1^). Similarly, FTIR spectroscopy could allow efficient distinction of murine iPSC cell line (M2A1) generated by reprogramming of murine embryonic fibroblasts (MEF). As can be seen in Fig. 1F, MEF exhibit a spectrum clearly different from the murine iPSC. By using this PLS-DA model, MEF spectra can be discriminated from the mIPS/mESC spectra with 100% sensitivity and 100% specificity (Table S2).The murine iPSC were found to exhibit a spectral signature similar to that of four murine ESC lines (D3, GS2, CJ7, R1) and were not separated by using the first principal components in the non-supervised PCA (data not shown), and in the supervised discriminant analysis PLS-DA (Fig. 1D), or in HCA. However, a PLS-DA model with 646 spectra of mIPSC and mESC allowed achieving a correct identification (MCC = 0.78) of 100 validation spectra using 6 factors explaining 99% of the spectral variance.

**Figure 2:**
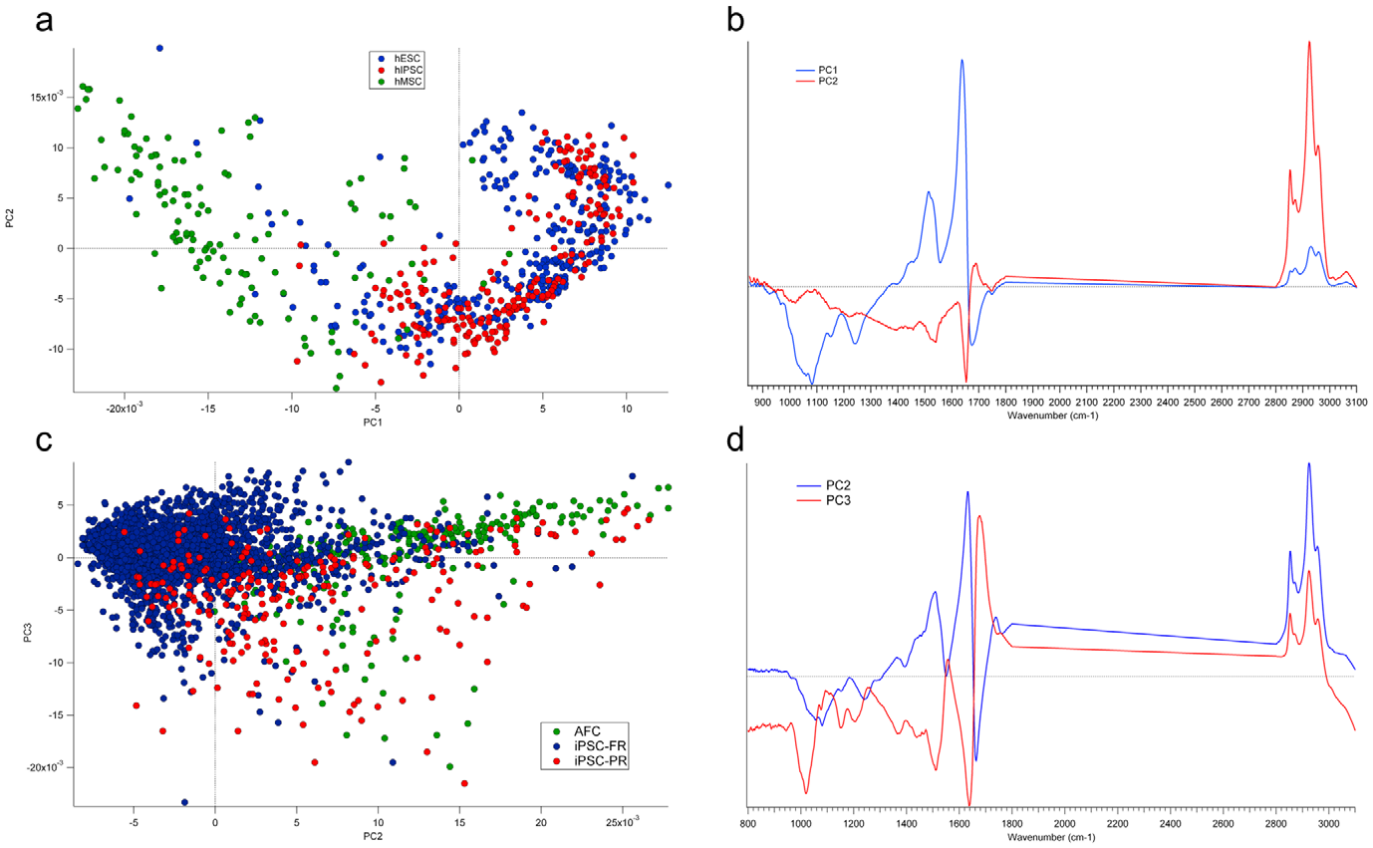
Identification of spectral signatures appearing during reprogrammation. (A) Comparison of spectral signature of MSC-H9 with IPSC-H9 and parental H9. Scoreplot of the PLS-DA show a distinct and separated spectra of MSC-H9 (green), iPSC-H9 (red) and H9 (blue). H9 and iPSC-H9 clustered together with a similar spectra. (B) Loading plot corresponding to the comparison of spectral signature of MSC-H9 with IPSC-H9 and parental H9. PC1 and PC2 show that the difference between MSC-H9 and ESC-H9/iPSC-H9 spectra lies in the lipid∶protein and in the protein∶nucleic acid contents. (C) Comparison of spectral signatures between Partially Reprogrammed (PR) and Fully Reprogrammed (FR) iPSC and AFC. Scoreplot of the PCA of PC2 versus PC3 (representing respectively 20 and 8% of the spectral variance) using the 800–1800 and 2800–3100 cm−1 ranges show a separation between iPSC-PR (PB08-PR, PB14, PB15, PB18) and iPSC-FR (PB03, PB04, PB08-FR, PB10, PB13) with a change in lipid and glycogen storage in the PR iPS. (D) Loading plot of the comparison of spectral signatures between Partially Reprogrammed (PR) and Fully Reprogrammed (FR) iPSC and AFC. PC2 and PC3 illustrate the chemical variability in protein, glycogen, lipid and nucleic acid contents between AFC and iPSC at different stages of the reprogrammation process.

These results prompted us to validate experimentally the generation of a “pluripotency spectra” signature by reprogramming strategies. To confirm these results, we devised a strategy to generate differentiated cells from the hESC H9 and to reprogram back these differentiated cells into the iPSC state by reprogramming factors (Figure S1). Mesenchymal stem cells (MSC) generated from H9, were characterized by phenotypic profiling and functional properties as described [9] (Figure S2) and then reprogrammed into iPSC. The iPSC generated from MSC-H9 (called hereafter as iPS-H9) exhibited all the markers of pluripotency similarly to ESC including generation of teratomas and had silenced the reprogramming transgenes Figure S3). The spectral signatures of individual ESC-H9, MSC-H9 and iPSC-H9 cells were recorded and their spectra analyzed using pattern recognition approaches (see Methods). The MSC-H9 spectra were found to be clearly different from those of the ESC and iPSC (Fig. 2A) and could be identified with 100% specificity and sensitivity by a discriminant analysis model with a MCC of 1 (Table S2). The MSC spectra feature higher lipid-to-protein and lower nucleic acid-to-protein ratios. Importantly, the iPSC-H9 and ESC-H9 spectra could not be separated by unsupervised methods (PCA, HCA) or supervised (PLS-DA) approaches using the first 3 PCs accounting for 85% of the variance. This shows that upon reprogramming, MSC-H9 have recovered the same chemical composition as the parental ESC-H9. However, a PLS-DA model capturing 99% of the spectral variance (8 factors) could separate the iPSC-H9 from the ESC-H9 with a MCC of 0.82 close to a perfect prediction (Table S2). As for murine cells, techniques using the most natural variance such as HCA, KMC and PCA could not discriminate accurately between the ESC and the iPSC. However, more sophisticated techniques such as PLS-DA were able to more reliably separate iPSC from ESC using a small percentage of the spectral variance (less than 1%) showing that FTIR spectroscopy can detect the small chemical differences that persist between those cells.

We next asked whether FTIR could discriminate *bona fide* iPSC from partially reprogrammed (PR) iPSC (PB14, PB15, PB18) characterized by the maintenance of transgene expression (Fig. S3) or the maintenance of the GFP expression in iPSC (PB08), the latter cell line being obtained by retroviral mediated transfer of pluripotency gene strategy using the GFP as a selection marker. The fully reprogrammed (FR) PB08-FR GFP-negative cells were compared with PB08-PR GFP-positive iPSC clones. In PCA the separation between PR and FR IPSCs spectra is carried by the second PC (Fig. 2D) which shows an increased lipid and glycogen storage in the IPSCs-PR . Most of iPSC-FR spectra were classified apart from those of iPSC-PR spectra. IPCS-PR were already engaged in the de-differentiation process and a continuum of spectral signatures existed between the differentiated and reprogrammed states.

Thus, FTIR microspectroscopy could also be used to separate out the candidate fully programmed iPSC clones from the partially programmed ones. A perfect prediction of iPSC-FR (100% sensitivity, 100% specificity and MCC = 1) could be achieved using a PLS-DA model with 10 factors and 98% of the variance (Table S2).

## Discussion

Several reports showed that infrared spectroscopy and microspectroscopy could be used to follow the differentiation state of adult stem cells [10], [11], [12] or ESC [13], [14], [15] but the reprogramming of differentiated cells into iPSC was never studied by this technology. Recently, a high-throughput dynamic single cell imaging technique describing iPSC generation has been reported [16] .The technique that we describe is complementary of this methodology and can be applied to large numbers of cells. We report here for the first time, that FTIR microspectroscopy could be used to follow the spectral signature of adult cells during reprogrammation. We demonstrate that a differentiated cell becoming a bona fide iPSC acquires the same metabolic features as ESC. The FTIR microspectroscopy could therefore be a promising technique for determining the metabolic fingerprint of individual ESC and iPSC in confluent colonies due to the high spectral quality and single cell resolution complemented by spectral pattern recognition techniques. FTIR spectroscopy and multivariate pattern recognition also managed to reliably identify the small chemical differences (less than 1% of the spectral variance) between iPSC and ESC using supervised method where class identity is known a priori.

The involvement of lipids in stem cell biology has been the subjects of several studies, showing high lipid rafts contents of adult and embryonic stem cells [17], [18]. Stem cell manipulation techniques with the use of synthetic lipids can modify the fate of cells grown in culture. For instance, recent studies suggested that ceramide was a critical cell-signaling factor for stem cell differentiation and cell polarity, two processes at the core of embryo development [17]. It has also been shown that ceramide was able to maintain the pluripotency of embryonic stem cells during culture without feeders on matrigel [19]. Our work demonstrate that FTIR microspectroscopy is an direct and robust method to follow the successfully reprogramming process and will be very useful in particularly in the case of integration-free iPSC generation. Finally, this technique can gain a wide application in the field of stem cell research by the availability of Raman infrared microscopy which can be accessed easily as compared to Synchrotron based FT-IR microspectroscopy.

## Materials and Methods

### ESC lines

Human embryonic stem cell lines H1, H9 were obtained from WiCell Research Institute, Madison, WI, (http://www.wicell.org), HUES3 from Howard Hughes Medical Institute (HUES Facility/Melton Laboratory), CL01, CL02, and CL03 from ESTeam Paris Sud, France (http://www.pfsc.fr). Human embryonic stem cells were used in this study under the agreements # RE07-008R provided by the French Biomedical Agency.

ESC lines were maintained in undifferentiated state by culture on a feeder layer of mitomycin C inactivated mouse embryonic fibroblasts in DMEM/F12 supplemented with 20% Knock Out serum replacer, 1 mM L-glutamine, 0.5% penicillin/streptomycin, 100 µM 2-mercaptoethanol, 10 ng/ml basic FGF (all of them from Invitrogen). Murine ES cell lines GS2 was kindly provided by A. Smith (ISCR, UK) and D3 CJ7, R1 from Inserm,Villejuif. Cells were maintained undifferentiated on gelatin-coated tissue culture plates in DMEM high glucose with glutamax (Invitrogen), supplemented with 15% ES-qualified FBS (Hyclone), 0,5% penicillin-streptomycin (Invitrogen), 100 µM 2-mercaptoethanol (Invitrogen) and 1000 U/ml recombinant murine LIF (ESGRO, Millipore).

### Generation of IPSC lines

Murine IPS cell line M2A1 was generated by transduction of 5×10^4^ CD1 mouse embryonic fibroblasts with ectopic retroviral particles carrying Oct4, Sox2, Klf4 and c-Myc vectors (Addgene). After 3–4 weeks, ES-like colonies appeared and were individually plucked. The individual clonal cells were then expanded and the M2A1 cell clone was further characterized for pluripotency and differentiation potential.

Human IPS cell lines were obtained by reprogramming mesenchymal stem cells derived from H9 hES cells (H9-IPS) (Figure S1).Adherent cells were characterized as MSC by flow cytometry exploring the expression of CD105, CD73, CD90, CD146, CD80, CD54 markers and the pluripotent markers Oct4, Sox2, Nanog and Lin28 (Figure S2A). Multipotency of MSC was tested for differentiation along the osteogenic, chondrogenic, and adipogenic lineages (Figure S2 B–D) using Functional Identification Kit (R&D Systems). Human iPSC were generated from amniotic fluid cells (PB03, PB04, PB08, PB09, PB10) and peripheral circulating endothelial progenitor cells (PB13). Amniotic fluid cells (AFC) and circulating endothelial progenitor cells (EPC) were used after informed consent of patients. For each human IPS cell line, 5×10^4^ to 1×10^5^ somatic cells were transduced with either 4 VSVG-pseudotyped lentivirus carrying Oct4, Sox2, Lin28 and Nanog vectors (Addgene) or 4 VSVG-pseudotyped retrovirus carrying Oct-4, Sox-2, Klf-4 and c-Myc vectors (Addgene). 4 to 6 weeks after transduction, undifferentiated hES-like colonies were manually picked and expanded on inactivated MEF in hESC medium. Colonies showed an ES cell–like morphology and were analyzed for the expression of pluripotent stem cell markers on a Macsquant flow cytometer with MacsQuantify software (Miltenyi Biotech). Samples of 2.5×10^5^ cells were stained with a combination of FITC conjugated mouse anti-human HESCA-1 antibody and PE conjugated mouse anti human SSEA-4 antibody (Flow Cellect Human ESC Surface Marker Characterization Kit, Millipore) according to the manufacturer instructions. For TRA 1–60 expression assessment, 1×10^5^ cells were stained in 10 microliters of PBS with 1 microliter of PE conjugated mouse anti human TRA 1–60 antibody or 1 microliter of unrelevant PE conjugated isotypic control (BD Biosciences). ESC and iPSC stained positively Tra1–60, HESCA-1 and SSEA-4 (Figure S3). Expression of exogenous transgenes was measured by Q-RT-PCR. The IPSC had repressed the transgenes and expressed the endogenous Oct4, Sox2, Nanog and Lin28 genes (Figure S4).

iPSC formed mature teratomas containing tissues of all three germ layers. Teratoma formation was induced by injection of 0.5×10^6^ to 3×10^6^ undifferentiated cells into the hind leg muscle of NOD/SCID mice. Tumors were resected 6 to 8 weeks after injection and immediately fixed in 4% paraformaldehyde in PBS before paraffin embedding. Tumor sections were then stained with hematoxylin and eosin before analysis for the presence of tissues representative of the three germ layers (ectoderm, mesoderm and endoderm).

The iPSC used in this study were in average at 20 passages and had normal karyotype without genomic instability regions as previously described [20]. ESC and iPSC lines were directly grown on IR reflective slides (MirrIR, Kevley Technologies) in the presence of Matrigel and mTeSR (Stem Cell Technologies) for infrared spectroscopy analysis. ESC Colonies are represented in Figure S5.

### Generation of partially reprogrammed iPSC

Partially reprogrammed IPSC were established during attempts to reprogram AFC. One iPSC (PB08-PR) were partially reprogrammed using MIGR-Oct4, MIGR-Sox2, MIGR-Klf4 and MIGR-c-Myc retroviral vectors and characterized by GFP-positive embryonic-stem-cell-like colonies. Three iPSC (PB14, PB15, PB18) derived from AFC by lentiviral Oct4, Sox2, Nanog and Lin28 vectors were also partially reprogrammed as they fail to repress ectopic transgene factors (Figure S4).

### Synchrotron radiation FTIR microspectroscopy

Primary AFC, MSC, ESC and iPS cell lines were grown on MirrIR slides for 3 to 4 days prior to analysis by *FTIR microspectroscopy*. Spectra were recorded at the SOLEIL synchrotron facility on the SMIS beamline which exploits the edge and bending radiations of a bending magnet. The synchrotron delivers 300 mA current in the 4/4 filling mode, in top-up mode for injection. Spectra were recorded in trans-reflexion on a Nicolet Continuum XL microscope (Thermo Fischer, Courtaboeuf, France) equipped with a 50×50 µm^2^ liquid nitrogen cooled MCT/A detector, a 32X/NA 0.65 Schwarzschild objective, XYZ motorised stage, and coupled to a Nicolet 5700 spectrometer (Thermo Fischer, Courtaboeuf, France) equipped with a Michelson interferometer, and a KBr beamsplitter. The confocal aperture was set at 12×12 µm^2^ which allowed measuring each individual cell within the colonies with an infrared spot size matching their size. The infrared spectrum of a stem cell is the overall signature of the cell composition and fingerprints its physiological state [21], [22], [23]. As proteins make up to 60–70% of the cell dry weight, the spectra of stem cells are dominated by the signal of the amide bands from the backbone of proteins, and show contributions from other major constituents: nucleic acids, lipids, and polysaccharides (Fig. 1A). For all cell lines, the spectra of 200 individual cells were collected for each of 3 independent cultures. Representative spectra of the different classes of cells are shown in Figure 1 and in Figure S5.

For individual cells, 128 to 256 scans per spectrum were coadded at 4 cm^−1^ resolution with Happ-Genzel apodization, and Mertz phase correction. All spectra were recorded between 800 and 4000 cm^−1^. To map entire colonies made of several thousands of cells, data collection rate was increased by two orders of magnitude by recording at 8 cm^−1^ resolution and 4–8 scans were coadded depending on colony size. Maps were recorded by raster scanning the colony with steps of 10 microm in X and Y, with the confocal aperture set at 12×12 microm^2^, resulting in the collection of approximately one spectrum per cell.

### Data analysis

Spectra of individual cells were analyzed by multivariate pattern recognition techniques in The Unscrambler (Camo Software AS, Oslo, Norway). Spectra were preprocessed before analysis: Extended Multiplicative Scattering Correction (EMSC) was applied between 800–1800 cm^−1^, and spectra were baseline corrected by subtracting a linear baseline between 1800 and 800 cm^−1^ to eliminate baseline shifts after EMSC correction. Alternatively, spectra were baseline corrected by subtracting a linear baseline in the 800–1800 cm^−1^ and in the 2800–3100 cm^−1^ ranges, and were then normalized to the same area in the concatenated ranges. In one case (iPS-PR, iPS-FR, AFC) the first derivative was used to increase the discriminant power of the method. The 800–1800 cm^−1^ range was found to be the most relevant to separate amniocytes foetal cells from IPSC and ESC, and IPSC from ESC, but the combined 800–1800 and 2800–3000 cm−1 range was more efficient for separating the differentiated cells (MSC and MEF) from their embryonic counterparts, and Fully Reprogrammed cells from Partially Reprogrammed cells.

Since cells were grown to confluence and formed a flat layer, little or no Mie scattering was observed. Spectra exhibiting Mie scattering (less than 1% of the spectra) were eliminated from the analysis.

The K-means clustering (KMC), Hierarchical Cluster Analysis (HCA), Principal Component Analysis (PCA), Partial Least Square Discriminant Analysis (PLS-DA) were carried out in the 800–1800 cm^−1^ range or in the concatenated 800–1800 cm^−1^, 2800–3100 cm^−1^ ranges.

PCA were carried out using preprocessed, mean centered data, 8 principal components were calculated using the NIPALS algorithm, and leverage correction was applied. Outliers were identified and eliminated using the Hotelling T2 method, and the residual versus leverage plot.

For PLS-DA, outliers were first eliminated by PCA. The remaining spectra were split in 2 sets: a calibration set and a smaller validation set containing a number of representative spectra. A PLS-DA model was created on the calibration set using the preprocessed, mean centered spectra. Random validation with the dataset divided in 20 segments was performed, and 10 factors were computed. A prediction was run on the validation set using 5 to 10 PLS-DA factors to use 98 to 99% of the explained spectral variance. Spectra were considered as identified when their prediction score was >0.5, and were considered as prediction outliers when deviation was above 0.5.

In order to evaluate the performances of the PLS-DA prediction, the sensitivity, specificity and the Matthews correlation coefficient (MCC) were computed according to the following equations:
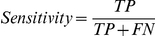


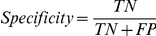


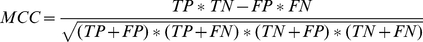
where TP is the number of true positives, TN the number of true negatives, FP the number of false positives, and FN the number of false negatives.

The MCC is a correlation coefficient used to evaluate the performance of binary classifications. It varies from ±1 to 0, +1 indicates a perfect identification, 0 is equal to the performance of a random classification. Only binary PLS-DA classification models were evaluated by the MCC.

The univariate analysis of the spectral maps was performed in OMNIC 7.4 (Thermo Fischer, Courtaboeuf, France) by plotting the area of one peak or the area ratio of two peaks. The multivariate analysis was performed in Cytospec 1.0 (Cytospec Inc., Boston, USA). by Hierchical Cluster Analysis HCA using D-values (based on Pearson correlation coefficient) and Ward's method.

## Supporting Information

Figure S1 **Schematic experimental model of H9 Reprogramming.** Pluripotency of H9 was lost by differentiation into Mesenchymal Stem Cell lineage. Pluripotency was re-induced by enforced expression of Oct4, Sox2, Nanog, Lin28 transgenes. ES-like colonies was picked-up and characterized and defined as iPSC-H9.
(TIF)

Figure S2 **Expression of pluripotency markers in ESC and iPSC by flow cytometry.** FACS analysis of SSEA-4 and HESCA-1 on H1, H9, HUES3,CL01, CL03, CL04 ESC (a–f), PB03, PB04, PB08, PB09, PB10, PB13 (g–l), MSC-H9 before reprogramming (m) and iPSC-H9 (n).
(TIF)

Figure S3 **Expression of reprogramming factors by RT-PCR.** Oct-4, Sox2, Nanog and Lin28 gene expressions were analysed in partially and fully reprogrammed iPSC and samples were normalized relative to an endogenous RNA control (TBP gene, which encodes TATA box-binding protein). For each factors, PCR were performed whit sets of primers recognizing total (endogenous and exogenous) and exogenous gene levels. Results expressed as N-fold differences in target gene expression relative to the TBP gene and termed “NTarget” were determined as NTarget = 2deltaCt sample, where the deltaCt value of the sample was determined by substracting the average Ct value of the target gene from the average Ct value of the TBP gene.
(TIF)

Figure S4 **Typical spectral cartography of hESC-H9 colonies.** H9 colonies were grown on slide and analyzed by FTIR for their composition of Amides and Sugars/Amides or lipids/Amide II. Ratios.
(TIF)

Figure S5 **Representative spectra of different cell types.** Spectra were offset for clarity. (A) Representative spectra of ESC-H9 cells illustrating the heterogeneity within one cell line. Nucleic Acid (NA)-rich (blue), NA-poor (red), and NA intermediate (green) spectra. (B) Average spectra of iPSC and AFC. Two representative spectra of iPSC are shown to illustrate the heterogeneity in the glycogen content of iPSC. (C) Average spectra of MEF (green), murine ESC (blue) and iPSC (red). Two spectra of iPSC and ESC are given to illustrate the similarity between different iPSC and ESC and the difference with MEF. (D) Average spectra of MSC-H9 (green), iPSC-H9 (red), and ESC-H9 (blue) cells. Two types of spectra are shown for ESC and iPSC and illustrate the similarity of the different iPSC and ESC spectral signatures.
(TIF)

Table S1 **Peak assignment for the infrared spectral signatures of stem cells.**
(DOCX)

Table S2 **Evaluation of the performances of PLS-DA model binary classifications.**
(DOCX)
