# Supplementary figures and images for: Identification of Spectral Modifications Occurring during Reprogramming of Somatic Cells
Source: PLoS One. 2012 Apr 13;7(4):e30743. doi: 10.1371/journal.pone.0030743 (PMC3326006; doi:10.1371/journal.pone.0030743)

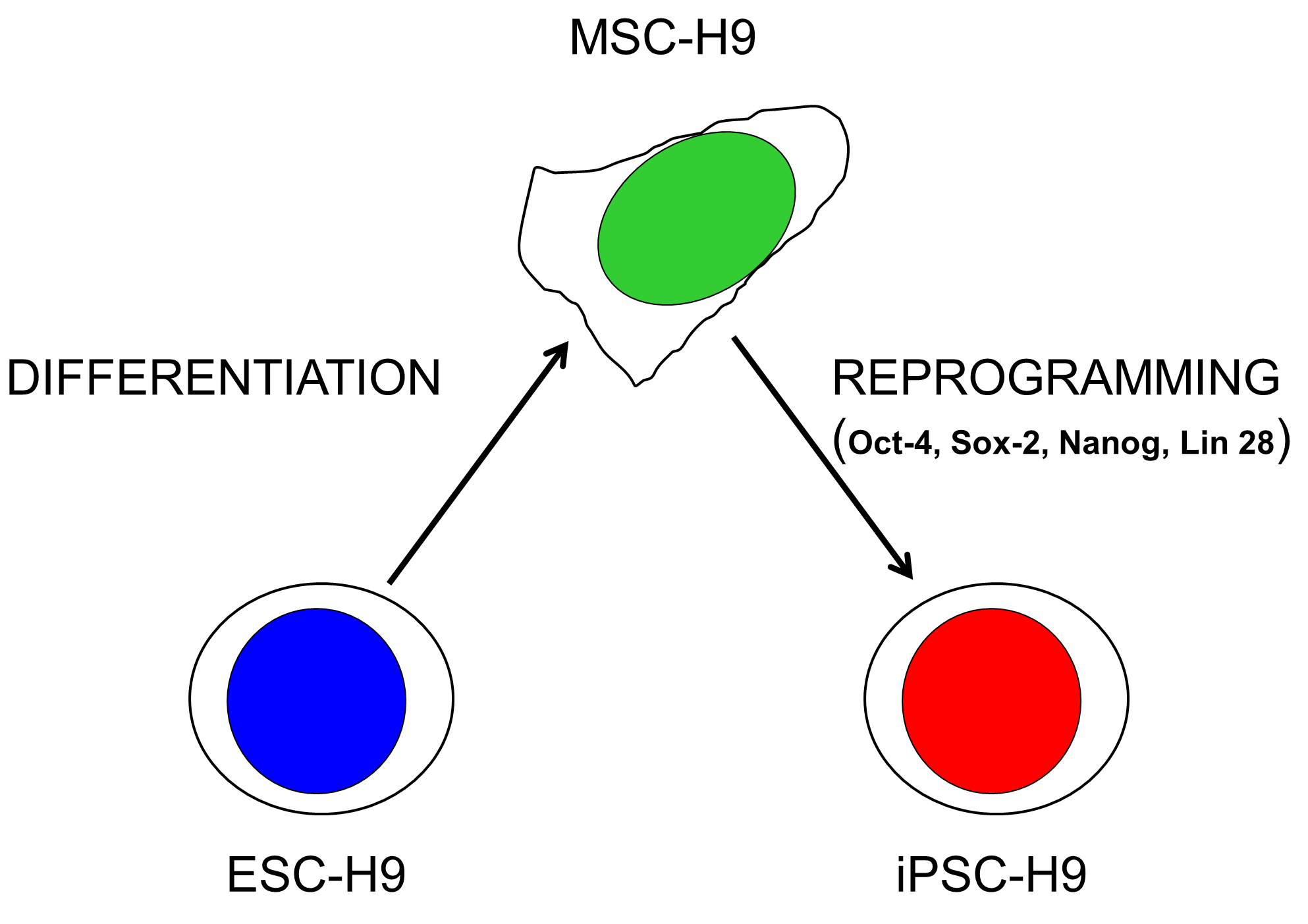

Supplement: Figure S1 — Schematic experimental model of H9 Reprogramming. Pluripotency of H9 was lost by differentiation into Mesenchymal Stem Cell lineage. Pluripotency was re-induced by enforced expression of Oct4, Sox2, Nanog, Lin28 transgenes. ES-like colonies was picked-up and characterized and defined as iPSC-H9. (TIF) [file pone.0030743.s001.tif]

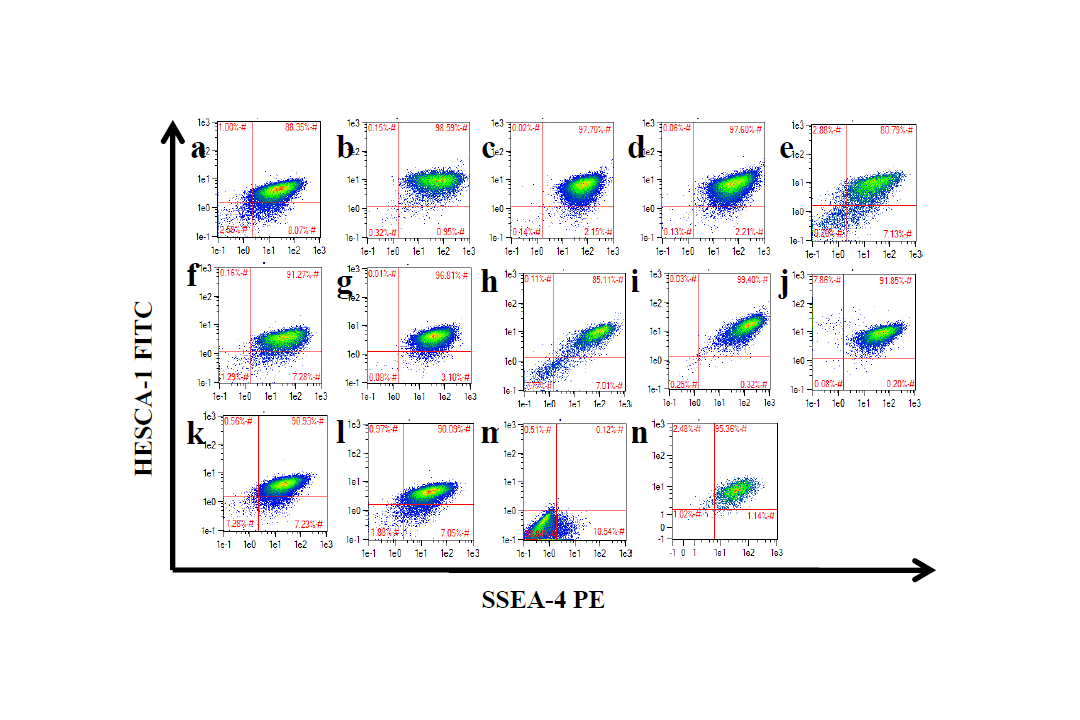

Supplement: Figure S2 — Expression of pluripotency markers in ESC and iPSC by flow cytometry. FACS analysis of SSEA-4 and HESCA-1 on H1, H9, HUES3,CL01, CL03, CL04 ESC (a–f), PB03, PB04, PB08, PB09, PB10, PB13 (g–l), MSC-H9 before reprogramming (m) and iPSC-H9 (n). (TIF) [file pone.0030743.s002.tif]

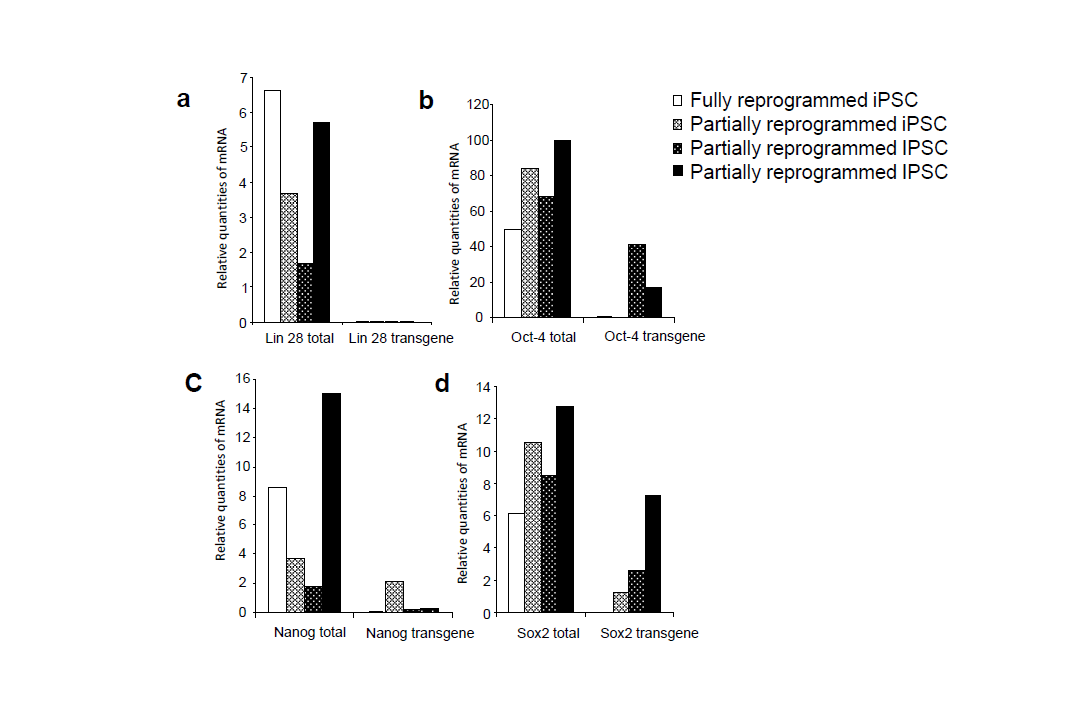

Supplement: Figure S3 — Expression of reprogramming factors by RT-PCR. Oct-4, Sox2, Nanog and Lin28 gene expressions were analysed in partially and fully reprogrammed iPSC and samples were normalized relative to an endogenous RNA control (TBP gene, which encodes TATA box-binding protein). For each factors, PCR were performed whit sets of primers recognizing total (endogenous and exogenous) and exogenous gene levels. Results expressed as N-fold differences in target gene expression relative to the TBP gene and termed “NTarget” were determined as NTarget = 2deltaCt sample, where the deltaCt value of the sample was determined by substracting the average Ct value of the target gene from the average Ct value of the TBP gene. (TIF) [file pone.0030743.s003.tif]

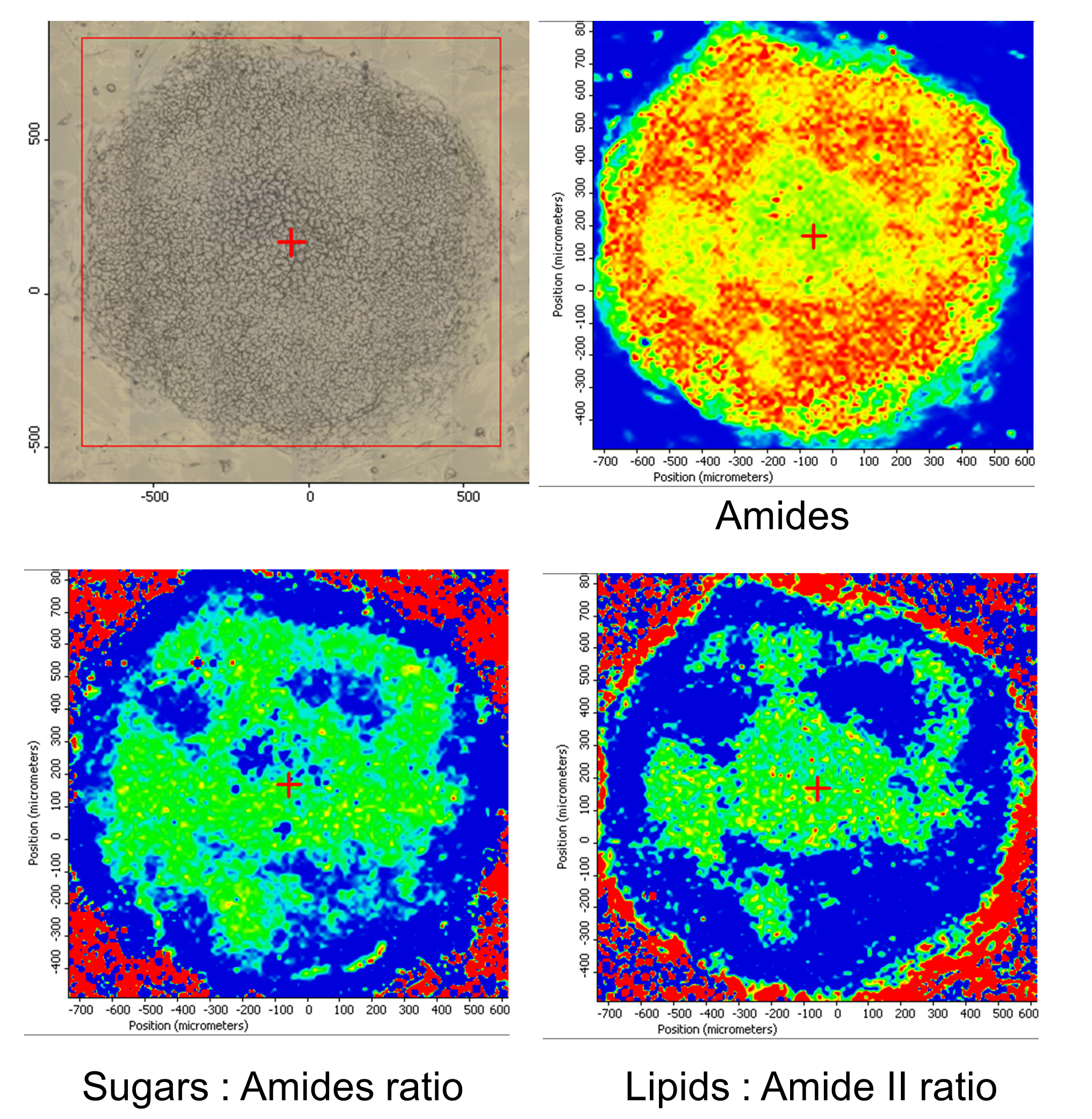

Supplement: Figure S4 — Typical spectral cartography of hESC-H9 colonies. H9 colonies were grown on slide and analyzed by FTIR for their composition of Amides and Sugars/Amides or lipids/Amide II. Ratios. (TIF) [file pone.0030743.s004.tif]

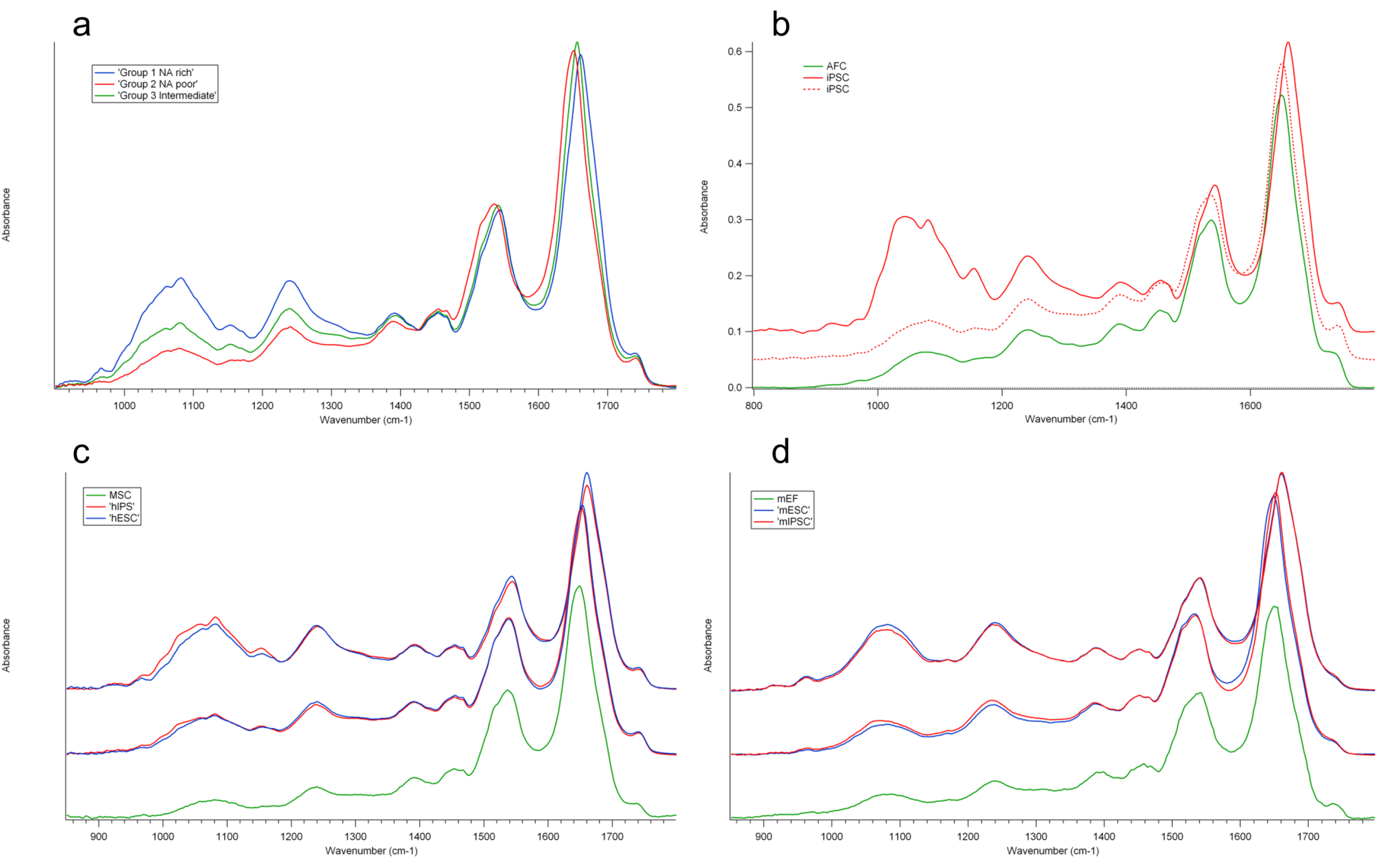

Supplement: Figure S5 — Representative spectra of different cell types. Spectra were offset for clarity. (A) Representative spectra of ESC-H9 cells illustrating the heterogeneity within one cell line. Nucleic Acid (NA)-rich (blue), NA-poor (red), and NA intermediate (green) spectra. (B) Average spectra of iPSC and AFC. Two representative spectra of iPSC are shown to illustrate the heterogeneity in the glycogen content of iPSC. (C) Average spectra of MEF (green), murine ESC (blue) and iPSC (red). Two spectra of iPSC and ESC are given to illustrate the similarity between different iPSC and ESC and the difference with MEF. (D) Average spectra of MSC-H9 (green), iPSC-H9 (red), and ESC-H9 (blue) cells. Two types of spectra are shown for ESC and iPSC and illustrate the similarity of the different iPSC and ESC spectral signatures. (TIF) [file pone.0030743.s005.tif]
