# Supplementary material for: Identification of Spectral Modifications Occurring during Reprogramming of Somatic Cells
Source: PLoS One. 2012 Apr 13;7(4):e30743. doi: 10.1371/journal.pone.0030743 (PMC3326006; doi:10.1371/journal.pone.0030743)
Supplement: Table S1 — Peak assignment for the infrared spectral signatures of stem cells. (DOCX) [file pone.0030743.s006.docx]

**Supplementary Table 1:**

**Peak assignment for the infrared spectral signatures of stem cells**

| **Band Position**  **(wavenumber cm-1)** | **Assignment** |
| --- | --- |
| 3600 | OH stretching |
| 3400 | NH stretching from proteins |
| 2800-3100 | CH stretching from proteins and lipids |
| 1740 | C=O stretching from lipid esters |
| 1645-1660 | Amide I from protein backbone |
| 1530-1540 | Amide II from protein backbone |
| 1450-1465 | CH bending |
| 1390-1400 | COO- |
| 1240 | Antisymmetric PO2— stretching from nucleic acids |
| 1080 | Symmetric PO2— stretching from nucleic acids |
| 980-1150 | CC, COC stretching from glycosidic linkage |
| 965 | Nucleic acids backbone |
