# Supplementary material for: Identification of Spectral Modifications Occurring during Reprogramming of Somatic Cells
Source: PLoS One. 2012 Apr 13;7(4):e30743. doi: 10.1371/journal.pone.0030743 (PMC3326006; doi:10.1371/journal.pone.0030743)
Supplement: Table S2 — Evaluation of the performances of PLS-DA model binary classifications. (DOCX) [file pone.0030743.s007.docx]

**Supplementary Table 2:**

**Evaluation of the performances of PLS-DA model binary classifications**

|  | **Fig 1b**  **iPSC vs ESC** | **Fig 1c**  **AFC vs iPSC** | **Fig 1d**  **MEF vs miPSC mESC** | **Fig 1d**  **mIPSC vs mESC** | **Fig 2a**  **MSC-H9 vs iPSC-H9 & ESC-H9** | **Fig 2a**  **iPSC-H9 vs ESC-H9** | **Fig 2b**  **iPS-FR vs iPS-PR** |
| --- | --- | --- | --- | --- | --- | --- | --- |
| **Calibration**  **set spectra** | **4260** | **439** | **760** | **646** | **682** | **426** | **505** |
| **Factors** | **8** | **8** | **6** | **6** | **8** | **8** | **10** |
| **Explained**  **variance** | **98%** | **98%** | **99%** | **99%** | **99%** | **98%** | **98%** |
| **Validation set spectra** | **265 ESC**  **240 iPSC** | **50 AFC**  **50 iPSC** | **100 ESC & iPSC**  **10 MEF** | **50 miPSC**  **50 mESC** | **56 MSC-H9**  **416 ESC-H9 & iPSC-H9** | **50 iPSC-H9**  **50 ESC-H9** | **50 iPS-PR**  **100 iPS-FR** |
| **TP** | **200** | **50** | **10** | **43** | **56** | **44** | **50** |
| **TN** | **169** | **50** | **100** | **46** | **416** | **47** | **100** |
| **FP** | **96** | **0** | **0** | **4** | **0** | **3** | **0** |
| **FN** | **40** | **0** | **0** | **7** | **0** | **6** | **0** |
| **Sensitivity** | **83%** | **100** | **100** | **86** | **100** | **88%** | **100** |
| **Specificity** | **63%** | **100** | **100** | **92** | **100** | **94%** | **100** |
| **MCC** | **0.48** | **1.00** | **1.00** | **0.78** | **1.00** | **0.82** | **1** |
